# Novel *SCARB2 *mutation in action myoclonus-renal failure syndrome and evaluation of *SCARB2 *mutations in isolated AMRF features

**DOI:** 10.1186/1471-2377-11-134

**Published:** 2011-10-27

**Authors:** Franziska Hopfner, Barbara Schormair, Franziska Knauf, Achim Berthele, Thomas R Tölle, Ralf Baron, Christoph Maier, Rolf-Detlef Treede, Andreas Binder, Claudia Sommer, Christian Maihöfner, Wolfram Kunz, Friedrich Zimprich, Uwe Heemann, Arne Pfeufer, Michael Näbauer, Stefan Kääb, Barbara Nowak, Christian Gieger, Peter Lichtner, Claudia Trenkwalder, Konrad Oexle, Juliane Winkelmann

**Affiliations:** 1 Institute of Human Genetics, Helmholtz Zentrum München-German Research Center for Environmental Health, Neuherberg, Germany; 2 Institute of Human Genetics, Klinikum rechts der Isar, Technische Universität München, Munich, Germany; 3 Department of Neurology, Klinikum rechts der Isar, Technische Universität München, Munich, Germany; 4 Sektion Neurologische Schmerzforschung und -therapie, Department of Neurology, Christian-Albrechts-Universität zu Kiel, Kiel, Germany; 5 BG University Hospital Bergmannsheil Bochum, Bochum, Germany; 6 Lehrstuhl für Neurophysiology, Medizinische Fakultät Mannheim der Ruprecht-Karls-Universität Heidelberg, Mannheim, Germany; 7 Department of Neurology, Universitätsklinikum Würzburg, Würzburg, Germany; 8 Department of Neurology, Friedrich-Alexander-Universität Erlangen-Nürnberg, Erlangen, Germany; 9 Department of Epileptology and Life & Brain Center, Universität Bonn, Bonn, Germany; 10 Department of Clinical Neurology, Medical University of Vienna, Vienna, Austria; 11 Department of Nephrology, Klinikum rechts der Isar, Technische Universität München, Munich, Germany; 12 Department of Medicine I, University Hospital Grosshadern, Ludwig-Maximilians-Universität München, Munich, Germany; 13 Zentrum für Nieren- und Hochdruckkrankheiten, Immenstadt and Oberstdorf, Germany; 14 Institute of Epidemiology, Helmholtz Zentrum München, German Research Center for Environmental Health, Neuherberg, Germany; 15 Paracelsus-Elena-Klinik, Center of Parkinsonism and Movement Disorders, Kassel, Germany; 16 Member of the German Research Network on Neuropathic Pain (DFNS

## Abstract

**Background:**

Action myoclonus-renal failure syndrome is a hereditary form of progressive myoclonus epilepsy associated with renal failure. It is considered to be an autosomal-recessive disease related to loss-of-function mutations in *SCARB2*. We studied a German AMRF family, additionally showing signs of demyelinating polyneuropathy and dilated cardiomyopathy.

To test the hypothesis whether isolated appearance of individual AMRF syndrome features could be related to heterozygote *SCARB2 *mutations, we screened for *SCARB2 *mutations in unrelated patients showing isolated AMRF features.

**Methods:**

In the AMRF family all exons of *SCARB2 *were analyzed by Sanger sequencing. The mutation screening of unrelated patients with isolated AMRF features affected by either epilepsy (n = 103, progressive myoclonus epilepsy or generalized epilepsy), demyelinating polyneuropathy (n = 103), renal failure (n = 192) or dilated cardiomyopathy (n = 85) was performed as high resolution melting curve analysis of the *SCARB2 *exons.

**Results:**

A novel homozygous 1 bp deletion (c.111delC) in *SCARB2 *was found by sequencing three affected homozygous siblings of the affected family. A heterozygous sister showed generalized seizures and reduction of nerve conduction velocity in her legs. No mutations were found in the epilepsy, renal failure or dilated cardiomyopathy samples. In the polyneuropathy sample two individuals with demyelinating disease were found to be carriers of a *SCARB2 *frameshift mutation (c.666delCCTTA).

**Conclusions:**

Our findings indicate that demyelinating polyneuropathy and dilated cardiomyopathy are part of the action myoclonus-renal failure syndrome. Moreover, they raise the possibility that in rare cases heterozygous *SCARB2 *mutations may be associated with PNP features.

## Background

Action myoclonus-renal failure syndrome (AMRF, OMIM 254900) is characterized by neurological symptoms such as progressive action myoclonus epilepsy (PME), dysarthria, ataxia, and generalized seizures. In addition, patients develop end-stage renal failure (RF) requiring dialysis and/or renal transplantation [1-4]. The syndrome is considered to be an autosomal-recessive disease related to loss-of-function mutations in *SCARB2 *which encode a lysosomal-membrane type 2 protein, a member of the CD 36 scavenger receptor-like protein family [5,6]. β-glucocerebrosidase (β-GC) trafficking requires SCARB2 protein binding for transfer from the endoplasmatic reticulum to the lysosome [6,7]. Scarb2^-/- ^mice have hydronephrosis, deficiency in glomerular filtration and peripheral demyelinating neuropathy but no epileptic sign [8]. They show normal cardiac development, but fail to show a hypertrophic response to increased blood pressure and develop dilated cardiomyopathy [9].

We examined an AMRF family with three affected siblings showing typical features [2]. Epileptic seizures and a loss of sensation in the feet occurred in one sister.

## Methods

### AMRF Family

We sequenced the *SCARB2 *gene in a previously described AMRF family [2,4]. Three of six children had AMRF and developed PME and renal failure within the first two decades of life. In addition to common AMRF features, all affected siblings also had demyelinating polyneuropathy (PNP). PNP was diagnosed before the patients developed renal failure [4]. For detection of peripheral neuropathy, electromyography (EMG) and nerve conduction studies (NCS) and/or skin punch biopsy were performed. In two siblings echocardiography revealed dilated cardiomyopathy (DCM) at the ages of 14 and 21. A sister of the affecteds had generalized seizures beginning at the age of 14 and a clinically diagnosed reduction of nerve conduction velocity in her legs.

### Patient Populations

We screened the following patient samples for mutations in *SCARB2*:

Epilepsy patients with PME or generalized epilepsy (GE): 28 unrelated German PME patients and 75 unrelated Austrian epilepsy patients. The German patients derived from a sample of 30 PME patients resembling Unverricht-Lundborg disease (nine females, 21 males, mean age: 26.8 ± 14.3 years) from which two were excluded since they were found to be siblings who had compound heterozygote *CSTB *mutations. The Austrian sample (40 female, 35 male, mean age: 29.3 years, range 18-68 years) was composed of three PME patients, seven juvenile epilepsy syndrome patients and 65 GE patients.

*Renal failure*: 192 unrelated German dialysis patients (76 female, 116 male, mean age: 66 ± 10.3 years).

*Cardiomyopathy*: 85 unrelated German patients with DCM (14 female, 71 male, mean age: 55 ± 13.2 years).

*Polyneuropathy*: 103 unrelated German patients with a clinical diagnosis of PNP (32 female, 71 male, mean age: 58 ± 13.5 years).

### Sequencing

Genomic DNA was extracted from peripheral blood using standard procedures for sequencing of 12 *SCARB2 *exons and 11 beta-glucosidase (*GBA*) exons, including the exon/intron boundaries. BigDye terminator chemistry 3.1 (ABI) and an ABI3730 sequencer were used. Primers were designed by Exon Primer [10] respectively Primer3plus [11] software. For sequence analysis the Staden software package was applied.

### RT-PCR

All 12 *SCARB2 *exons were screened for deletions and duplications by real-time polymerase chain reaction (RT-PCR) in all members of the AMRF family and in PNP patients using an ABI7900HT system and a SYBR Green I detection dye. Gene dosage was determined using the ΔCT method. To control variation of DNA concentration and PCR efficiency *BNC1 *was used as a reference gene. The mean gene dosage of a healthy control was used for calibration. All samples were taken in duplicates.

### *SCARB2 *Mutation Screening

For mutation screening in the selected patient population, i.e. epilepsy patients, renal failure patients, cardiomayopathy patients and polyneuropathy patients high resolution melting curve analysis of amplicons, spanning the 12 exons and splice junctions of *SCARB2*, was performed. PCR was run in a 5 μl reaction mixture containing: Thermo Scientific High Performance Buffer with the concentration 1x, 2.5 mM MgCl_2_, 0.2 mM dNTPs, 0.05 U/μl Taq DNA Polymerase, Primer 0.4 μM each, DNA-binding dye LC-Green-Plus (Idaho Technology Inc., Salt Lake City, UT) with the concentration 0.5x, 2.8 μl H_2_O_HPLC _and 8 ng of genomic DNA.

To facilitate heteroduplex formation and in order to detect homozygous variants, reactions were spiked with wild type genomic DNA (1: 20 titration). The mixture was covered with a mineral oil overlay to prevent evaporation during heating on the LightScanner. Melting data were analysed using the LightScanner Software Call-IT v1.1 (Idaho Technology). The presence of a mutation was revealed by an aberrant melting curve. Samples flagged as potential variants were subjected to DNA sequencing.

All experimental research was done in compliance with the Helsinki Declaration. This research was conducted retrospectively therefore yielding minimum consequences to the subjects. We protect the privacy of research subjects and the confidentiality of their personal information to minimize the impact of the study on their physical, mental and social integrity.

## Results

### Gene analysis of the AMRF family

**Figure 1:**
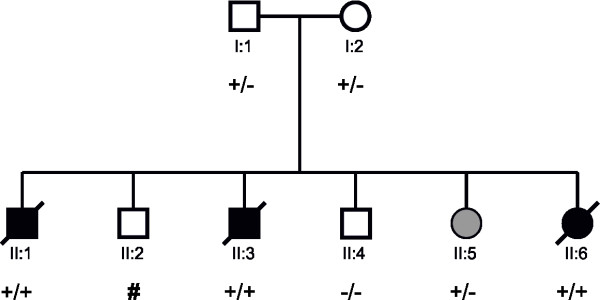
**Pedigree of the AMRF family with mutation status**. Black symbol: AMRF. Grey symbol: generalized epilepsy and reduction of nerve conduction velocity in the feet. +/+: homozygous mutation, +/-: heterozygous mutation. Symbol #: no information available about genotype status.

**Figure 2:**
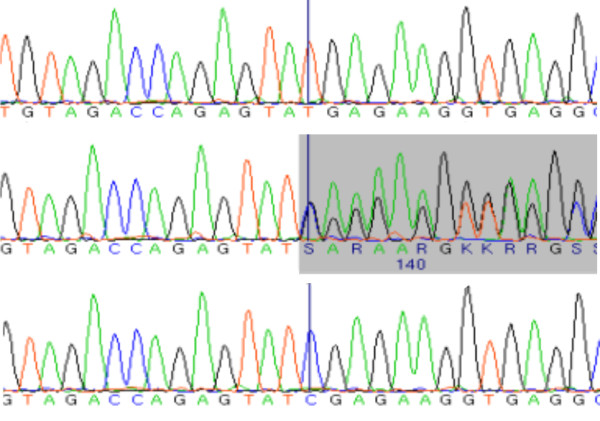
***SCARB2 *sequence in the AMRF family**. Top row, homozygous c.111delC mutation of patient II:3. Middle row, sequence of heterozygous carrier II:5. Bottom row, wild type sequence.

In the patients II:1, II:3 and II:6 (Figure 1, Table 1, Table 2) we identified a homozygous 1 bp-deletion (c.111delC) in exon1 (Figure 2) resulting in frameshift and premature stop after 17 bp. In patients I:1, I:2, we identified the same deletion as being heterozygous and in patient II:4 we could not detect any mutation. There is no information available about genotype status of patient II:2. Patient II:5 carried the same deletion in a heterozygous state. Sequencing of *GBA *in all family members did not reveal any mutation in any member of this family.

**Table 1:** Onset of neurological features [yrs] in members of the AMRF pedigree depicted in Figure 1 and Figure 2

patient [pedigree number]	sex	age of onset	action myoclonus	tonic clonic seizures	ataxia	demyelinating polyneuropathy	hearing impairment	age at death [yrs]
II:1	m	14	14	20	14	+	NA	31

II:6	f	20	20	20	20	+	26	34

II:3	m	20	26	32	20	+	NA	38

II:5	f	14	-	14	-	unclear	-	alive

If known, the age of onset [yrs] of a feature is indicated; "+" = present but age of onset unknown; "-" = absent; "NA" = not assessed.

**Table 2:** Onset of renal and cardiovascular features [yrs] in members of the AMRF pedigree depicted in Figure 1 and Figure 2

patient [pedigree number]	sex	age of onset	dialysis	renal trans-plan-tation	dilated cardio-myopathy	hypertension	age at death [yrs]
II:1	m	14	18	-	< 14	+	31

II:6	f	20	22	-	< 21	+	34

II:3	m	20	26	31	-	+	38

II:5	f	14	-	-	-	+	alive

If known, the age of onset [yrs] of a feature is indicated; "+" = present but age of onset unknown; "-" = absent; "NA" = not assessed.

### Screening for *SCARB2 *mutations in GE, RF, and DCM patient population

*SCARB2 *high resolution melting analysis of the GE, RF, and DCM patients did not reveal any case of apparent mutation.

### Screening for *SCARB2 *mutations in PNP patient population

**Figure 3:**
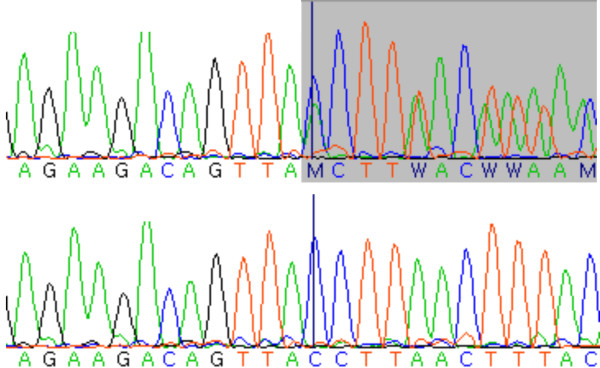
***SCARB2 *sequence in a PNP patient**. Top row, heterozygous mutation c.666delCCTTA. Bottom row, wild type sequence.

The 103 unrelated German PNP patients have been diagnosed by clinical investigation as well as standardized quantitative sensory testing (QST). Among the 103 PNP patients we identified two individuals carrying a heterozygous frameshift mutation in an exonic region of *SCARB2*, i.e. c.666delCCTTA respectively p.Y222XfsX in exon5 (Figure 3). This mutation has been described before in a patient with PME [12]. Neither this mutation nor the mutation detected in the AMRF family were found in 1452 normal chromosomes of the general population indicating that these are rare variants with a frequency below 0.1%. Controls were of European descent and were recruited from the KORA S3/F3 and S4 surveys, i.e. from the general population living in or near the city of Augsburg, Germany. KORA procedures and samples have been described previously [13].

## Discussion

The phenotype of the action myoclonus-renal failure syndrome (AMRF) is variable [2]. Some patients do not show evidence of the classic combination of epilepsy and renal failure. Homozygous and compound heterozygous mutations of *SCARB2 *have been identified in patients who had only progressive myoclonus epilepsy (PME) but no renal failure (RF) [12]. On the other hand, the spectrum of features may possibly be more extended than currently assumed. Here, we report an AMRF family with a novel 1 bp deletion (c.111delC). Of the three affected homozygotes in this family, two had cardiomyopathy (DCM) and all three had demyelinating polyneuropathy (PNP) already before the onset of renal failure. Our findings suggest that DCM and PNP are facultative features of the AMRF phenotype. The detection of PNP in AMRF syndrome is in keeping with observations on scarb2 ^-/- ^mice where SCARB2-deficiency was found to be associated with upregulation of lysosomal enzymes. This is concomitant with a downregulation of peripheral myelin proteins, accumulation of abnormal inclusions in the outer cytoplasmic zone of Schwann cells, massive loss of peripheral myelin proteins, onion bulbs and other myelin abnormalities, and secondary axonal degeneration, restricted to the peripheral nervous system [8].

A 27-year-old, heterozygous sibling of patients II:1, II:2, II:3, II:4, II:6 had generalized seizures beginning at the age of 14. She also additionally exhibited a reduction of nerve conduction velocity. However, the heterozygous mutation was associated with a detectable AMRF feature only in this sibling, whereas the other heterozygotes in the family (parents, other sister) were by history asymptomatic. This observation suggested that the potential association cannot be strong. To exclude a synergistic effect of two compound heterozygous mutations in the same lysosomal pathway, we additionally sequenced the 11 beta-glucosidase (*GBA*) exons. No *GBA *mutation was found. The phenotypical effect of heterozygous *SCARB2 *mutations has been discussed before in the case of a patient with myoclonus epilepsy and isolated proteinuria [14]. Moreover, earlier studies on families affected by myoclonus epilepsy of Unverricht and Lundborg (EPM1; *CSTB *mutations; OMIM 254800) reported mild symptoms in heterozygous carriers of the disease allele [15,16]. In keeping with the latter observation, features of the EPM1 phenotype were described in cstb^+/- ^mice [17]. Similarly, screening of *scarb2*^+/- ^mice for AMRF features may help to elucidate the impact of heterozygous *SCARB2 *mutations.

Therefore, we examined the possibility that the frequency of *SCARB2 *mutations may be increased in patients with isolated features of AMRF. To further examine the possible relationship between heterozygous mutations in *SCARB2 *and isolated AMRF signs such as generalized epilepsy (GE) (n = 75), progressive action myoclonus epilepsy (PME) (n = 28), renal failure (RF) (n = 192), polyneuropathy (PNP) (n = 103), and dilated cardiomyopathy (DCM) (n = 85), respective patient samples were scrutinized for putative mutations. We identified two heterozygous PNP patients carrying the *SCARB2 *mutation p.Y222XfsX located within the highly conserved β-GC binding domain [7]. Before, this frameshift mutation has been identified as being homozygous in a PME patient [12]. In the 103 PNP patients electrophysiological data on the nerve conduction study is not available. However, histopathological analysis of a nerve biopsy taken from one of the two patients carrying the p.Y222XfsX mutation showed evidence of demyelinating PNP. We have not performed any mutational screening of our DCM patients in sarcomeric proteins. None of the other monosymptomatic patients had a *SCARB2 *mutation. Thus, our findings are not in line with a recent report of high frequency of homozygous or compound heterozygote *SCARB2 *mutations in patients with isolated PME (clinically resembling Unverricht-Lundborg disease but being negative for CSTB mutations) [12]. However, the frequency of *SCARB2 *mutations in PME patients may differ between different populations.

Note added in proof: A recent report on a patient with PME and a demyelinating neuropathy affirmed our finding that polyneuropathy belongs to phenotype spectrum of *SCARB2 *mutation [18,19].

## Conclusions

We conclude that *SCARB2 *mutations may possibly contribute to the genesis of isolated AMRF-like features in rare cases, but do not appear to be a major cause of these features in the general population. To assess these findings further populations with PNP would have to be examined to see how frequently heterozygote *SCARB2 *mutations can explain a PNP.

## Competing interests

The authors declare that they have no competing interests. The authors disclose that they have been influenced by their personal or financial relationship with other people or organizations concerning interpretation of data or presentation of information.

These data were presented at the GfH-Jahrestagung 2011/03/16-18, in Regensburg, Germany.

### Financial disclosure related to research covered in this article

Hopfner F: intramural funding

Schormair B: intramural funding

Knauf F: intramural funding

Berthele A: German Ministry of Education and Research (BMBF), Grant support to the German Research Network on Neuropathic Pain (DFNS) **(Grants)**

Tölle TR: German Ministry of Education and Research (BMBF), Grant support to the German Research Network on Neuropathic Pain (DFNS) **(Grants)**

Baron R: German Ministry of Education and Research (BMBF) **(Grants)**, Pfizer Genzyme Grünenthal, Mundipharma, Allergan, Sanofi Pasteur, Medtronic, Eisai, UCB BioSiences, Lilly, Boehringer Ingelheim, Astellas, Novartis **(Consultancy, speakers bureaus)**, Pfizer Grünenthal **(Board membership)**, Pfizer Genzyme Grünenthal **(Grant)**

Maier C: none

Treede RD: German Ministry of Education and Research (BMBF), Deutsche Forschungsgemeinschaft, Dr. Kade. Consultant or speaker: Allergan, Astellas, AWD, Boehringer Ingelheim, Galderma, GlaxoSmithKline, Grünenthal, Lilly, Merz, Nycomed, Pfizer, UCB

Binder A: German Ministry of Education and Research (BMBF) **(Grants)**, Grünenthal, Pfizer, Genzyme **(Grants)**, Pfizer, Grünenthal, Astellas **(speakers bureaus)**, Pfizer, Grünenthal, Astellas Travel/accommodations/meeting expenses unrelated to activities listed **(other)**, Pfizer Payment for development of educational presentations **(other)**

Sommer C: none

Maihöfner C: German Ministry of Education and Research (BMBF) **(Grants)**, Grant support to the German Research Network on Neuropathic Pain (DFNS), Allergan, Pfizer, Bionorica **(Consultancy)**, German Research Foundation (DFG) (Grant)

Kunz W: German Ministry of Education and Research (BMBF) **(Grants)**

Zimprich F: none

Heemann U: none

Pfeufer A: intramural funding

Näbauer M: none

Kääb S: none

Nowak B: none

Gieger C: none

Lichtner P: Helmholtz Zentrum München (Employment) (**other)**

Trenkwalder C: BI, UCB **(Board membership)**, Associate Prof. of Neurology University of Goettingen, Medical Director of the Paracelsus-Elena-Clinic Kassel, Germany (Employment) **(other)**

Oexle K: none

Winkelmann J: National, UCB Advisory board RLS **(Consultancy)**, DFG Grant, Genetics of Iron metabolism, German RLS patient foundation **(Grants)**, Honoraria for lectures for UCB and Boeringer Ingelheim (Payment for lectures including service on speakers bureaus) **(other)**

## Authors' contributions

### Research project

Conception: WJ, OK, HF

Organization: WJ, HF

Execution: HF, SB, KF

Clinical support: TC, NB

Material support: BA, TTR, KW, BR, MC, TRD, BA, SC, MC, ZF, HU, PA, NM, KS, GC

### Manuscript Preparation

Writing of the first draft: HF, OK, WJ

Review and Critique: WJ, OK, LP, SB, KF, HU

All authors read and approved the final manuscript.

## Pre-publication history

The pre-publication history for this paper can be accessed here:

http://www.biomedcentral.com/1471-2377/11/134/prepub
